# Association of race and in‐hospital outcomes following acute pulmonary embolism: A retrospective cohort study

**DOI:** 10.1002/clc.24055

**Published:** 2023-05-31

**Authors:** Alexander E. Sullivan, Carlos E. Barbery, Tara Holder, Cynthia L. Green, Manesh R. Patel, Kevin L. Thomas, W. Schuyler Jones

**Affiliations:** ^1^ Department of Medicine Vanderbilt University Medical Center Nashville Tennessee USA; ^2^ Division of Cardiovascular Medicine, Department of Medicine University of Pennsylvania Philadelphia Pennsylvania USA; ^3^ Duke Clinical Research Institute Duke University School of Medicine Durham North Carolina USA; ^4^ Department of Biostatistics and Bioinformatics Duke University School of Medicine Durham North Carolina USA; ^5^ Division of Cardiology Duke University School of Medicine Durham North Carolina USA

**Keywords:** mortality, pulmonary embolism, racial disparities, venous thromboembolism

## Abstract

**Background:**

Racial disparities in health care are well established, with Black patients frequently experiencing the most significant consequences of this inequality. Acute pulmonary embolism (PE) is increasing in incidence and an important cause of morbidity and mortality in the United States, but little is known about racial disparities in the inpatient setting.

**Hypothesis:**

Black and White patients admitted with acute PE will have different in‐hospital outcomes.

**Methods:**

All PE patients from January 1, 2016 to June 30, 2017 were retrospectively identified using ICD‐10 codes. Data were abstracted by manual chart review for all image‐confirmed PEs.

**Results:**

A total of 782 patients with acute PE were identified, of which 319 (40.8%) were Black and 463 (59.2%) were White. Black patients had higher BMI (median [Q1–Q3]: 30.3 [25.4–36.6] vs. 29.3 [24.5–33.8] kg/m^2^, *p* = .017), were younger (61 [48–74] vs. 67 [54–75] years, *p* = .001), and were more likely to have a history of heart failure (16.0 vs. 7.1%, *p* < .001), while White patients had higher rates of malignancy (46.9 vs. 34.5%, *p* = .001) and recent surgery (29.6 vs. 18.2%, *p* < .001). Black patients were more likely to receive systemic thrombolysis (3.1% vs. 1.1%, *p* = .040), while White patients had numerically higher rates of surgical embolectomy (0.3% vs. 1.1%, *p* = .41). No difference in inpatient mortality was observed; however, Black patients had longer hospital length of stay (5.0 [3–9] vs. 4.0 [2–9] days, *p* = .007) and were more likely to receive warfarin (23.5 vs. 12.1%, *p* < .001).

**Conclusions:**

Similar in‐hospital mortality rates were observed in Black and White patients following acute PE. However, Black patients had longer hospital stays, higher warfarin prescription, and fewer traditional PE‐related risk factors.

## Graphical abstract

During index hospitalization for acute pulmonary embolism (PE), Black and White patients had similar PE severity with no difference in all‐cause mortality. However, malignancy, trauma, and recent surgery were more common risk factors in White patients, while Black patients more often had heart failure, diabetes, and kidney disease. Black patients had longer hospitalizations and were more frequently discharged on warfarin. CKD, chronic kidney disease; ICU, intensive care unit; LMWH, low molecular weight heparin; PE, pulmonary embolism; tPA, tissue plasminogen activator.

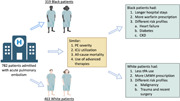

## 1 INTRODUCTION

Racial disparities are well documented in health care. Within cardiovascular disease, Black Americans have higher rates of morbidity and mortality following diagnosis of coronary artery disease, atrial fibrillation, aortic stenosis, and sudden cardiac death, but are less likely to receive guideline‐directed medications and procedures.^1^, ^2^, ^3^, ^4^, ^5^, ^6^, ^7^

These disparities are especially important in the care of venous thromboembolism (VTE), specifically acute pulmonary embolism (PE). This common condition affects nearly 1 million Americans annually and is the third leading cause of cardiovascular mortality.^8^, ^9^, ^10^ Black patients are nearly twice as likely to be hospitalized for acute PE and suffer 50% higher rates of 30‐day mortality.^11^, ^12^, ^13^ Black patients are also more likely to experience complications from treatment, including bleeding and inadequate anticoagulation, when compared to other races.^14^, ^15^, ^16^

Health disparities and outcomes following index hospitalization for acute PE remain under investigation. In this study, we aimed to: (1) describe the characteristics of Black and White patients hospitalized for management of acute PE, (2) assess differences by race in performance of risk‐stratifying diagnostic tests and referral for advanced therapies, and (3) describe the association between race and outcomes, including all‐cause in‐hospital mortality, discharge to hospice, and both intensive care unit (ICU) and hospital length of stay.

## 2 METHODS

Methods are described in detail in a previously publication by Holder et al.^17^

### 2.1 Patient identification

Our large health system is comprised of approximately 1500 inpatient beds across one academic hospital and two community hospitals. ICD‐10 codes for PE were utilized to identify an initial cohort of patients who presented from January 1, 2016 to June 30, 2017. An electronic health record (EHR) database was queried for demographic, treatment, and outcome information during initial hospitalization. This study was IRB exempt and no informed consent was required.

### 2.2 Inclusion/exclusion criteria

Inclusion and exclusion criteria are described in the prior study.^17^ Patients ≥18 years of age with imaging‐confirmed acute PE who identified as Black or non‐Hispanic White were included. Patients with any other thromboembolic event including, but not limited to, fat embolism, air embolism, septic embolism, chronic thromboembolic pulmonary hypertension were excluded. Patients without imaging confirmed PE were also excluded.

### 2.3 Chart review

Upon confirmation of PE diagnosis, baseline characteristics (e.g., age, sex, body mass index [BMI], and race/ethnicity), comorbidities (e.g., type 2 diabetes mellitus [DM], hypertension [HTN], congestive heart failure [CHF], chronic kidney disease [CKD]), PE risk factors (e.g., cancer history [including both active and previously treated malignancy excluding skin cancers], major surgery or trauma within 3 months of PE diagnosis, hypercoagulable states, hormonal therapy, pregnancy, tobacco use), presenting symptoms (e.g., chest pain, shortness of breath, syncope, presyncope, cough, hemoptysis, and leg swelling), vital signs, initial cardiac markers (e.g., troponin and brain natriuretic peptide [BNP]), imaging results (e.g., computed tomography [CT], echocardiogram, and ventilation/perfusion [V/Q] scan), use of invasive therapy (e.g., catheter directed therapy and systemic thrombolysis), discharge location (e.g., home, skilled nursing facility), as well as ICU and hospital length of stay (LOS) were manually abstracted. Definitions of all variables and details regarding chart review are available in the prior study.^17^

### 2.4 Outcomes

The primary outcome was the association between race and the composite endpoint of all‐cause mortality during index hospitalization for PE and discharge to hospice. Other outcomes of interest included the association between race and hospital utilization defined as ICU admission, ICU LOS, and hospital LOS.

### 2.5 Statistical analysis

Continuous data are presented using the median with 25th and 75th percentiles (Q1–Q3), while categorical data are shown using counts and percentages of non‐missing data. Black and White patient groups were compared using the Wilcoxon rank‐sum test for continuous variables and chi‐square or Fisher's exact test for categorical variables. Group comparisons of baseline characteristics, PE risk factors, ordered diagnostic tests and their results, ICU admission and LOS, treatment modality, hospitalization LOS, in‐hospital mortality, as well as the composite of all‐cause mortality and discharge to hospice were performed. ICU and hospital LOS were defined as the time to discharge or death, whichever occurred first.

Multivariable generalized linear models were used to evaluate the association of race with ICU admission and LOS, hospital LOS, warfarin use and the composite of in‐hospital mortality and/or discharge to hospice while controlling for baseline risk factors found imbalanced between groups (age, sex, BMI, HTN, CHF, DM, CKD, cancer, and prior surgery/trauma). Linearity assumptions of continuous variables were verified using regression splines. Results are presented as the odds ratio (OR) or incidence rate ratio (IRR) with 95% confidence interval (CI) dependent on logistic or negative‐binomial regression model, respectively.

All analyses were conducted using SAS version 9.4 (SAS Institute Inc.), and a *p*‐value < .05 was considered statistically significant.

## 3 RESULTS

### 3.1 Patient demographics

**Figure 1:**
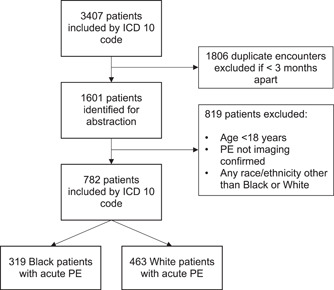
Flow diagram of cohort identification. Flow diagram of the 3407 initially identified patients along with application of exclusion and inclusion criteria. PE, pulmonary embolism.

Initially, 3407 unique patient encounters were identified. After excluding duplicate encounters and repeat presentations within 3 months of PE diagnosis, 1601 were eligible for chart review and 782 total patients met inclusion criteria. Of the 782 patients, 319 (40.8%) patients were Black and 463 (59.2%) were White (Figure 1). Black patients were younger (median [Q1–Q3] age: 61 [48–74] vs. 67 [54–75] years, *p* = .001), predominantly female (58.3% vs. 51.0%, *p* = .043) and had a higher BMI (30.3 [25.4–36.6] vs. 29.3 [24.5–33.8] kg/m^2^, *p* = .017) compared to White patients (Table 1). Black patients were more likely to have hypertension (62.1 vs. 53.8%, *p* = .021), congestive heart failure (16.0 vs. 7.1%, *p* < .001), diabetes mellitus (25.7 vs. 13.4%, *p* < .001) and chronic kidney disease (14.7 vs. 6.5%, *p* < .001). White patients were more likely to have cancer (46.9 vs. 34.5%, *p* = .001) and a recent encounter for surgery or trauma (29.6 vs. 18.2%, *p* = .001) (Table 1).

**Table 1:** Baseline demographics and clinical characteristics by race.

	Black (*N* = 319)	White (*N* = 463)	Total (*N* = 782)	*p* value
Demographics				
Age (years)	61 [48–74]	67 [54–75]	66 [51–75]	.001
Age >65	136 (42.6%)	256 (55.3%)	392 (50.1%)	.001
Sex, male	133 (41.7%)	227 (49.0%)	360 (46.0%)	.043
BMI (kg/m^2^)	30.3 [25.4–36.6]	29.3 [24.5–33.8]	29.6 [24.9–34.9]	.017
Comorbidities				
Hypertension	198 (62.1%)	249 (53.8%)	447 (57.2%)	.021
Hyperlipidemia	70 (21.9%)	115 (24.8%)	185 (23.7%)	.35
CAD	32 (10.0%)	53 (11.4%)	85 (10.9%)	.53
CHF	51 (16.0%)	33 (7.1%)	84 (10.7%)	<.001
COPD	29 (9.1%)	54 (11.7%)	83 (10.6%)	.25
Diabetes	82 (25.7%)	62 (13.4%)	144 (18.4%)	<.001
Stroke	9 (2.8%)	7 (1.5%)	16 (2.0%)	.20
Kidney disease	47 (14.7%)	30 (6.5%)	77 (9.8%)	<.001
PE risk factors				
Cancer history	110 (34.5%)	217 (46.9%)	327 (41.8%)	.001
Surgery/trauma	58 (18.2%)	137 (29.6%)	195 (24.9%)	<.001
Hypercoagulable	18 (5.6%)	20 (4.3%)	38 (4.9%)	.40
Hormone therapy	14 (4.4%)	17 (3.7%)	31 (4.0%)	.61
Pregnancy	6 (1.9%)	2 (0.4%)	8 (1.0%)	.069
Tobacco use	173/310 (54.2%)	247/448 (53.3%)	420/758 (53.7%)	.86
Presenting symptoms				
Dyspnea	171 (53.6%)	262 (56.6%)	433 (55.4%)	.41
Chest pain	106 (33.2%)	111 (24.0%)	217 (27.7%)	.005
Syncope/Pre‐syncope	32 (10.0%)	33 (7.1%)	65 (8.3%)	.15
Limb pain	28 (8.8%)	40 (8.6%)	68 (8.7%)	.95
Limb swelling	40 (12.5%)	51 (11.0%)	91 (11.6%)	.51
Hemoptysis	10 (3.1%)	8 (1.7%)	18 (2.3%)	.20
Cough	50 (15.7%)	47 (10.2%)	97 (12.4%)	.021
Fatigue	35 (11.0%)	36 (7.8%)	71 (9.1%)	.13
Hemodynamics				
SBP < 90	29 (9.1%)	44 (9.5%)	73 (9.3%)	.85
HR > 100	203 (63.6%)	275 (59.4%)	478 (61.1%)	.23
SpO_2_ < 90%	153 (48.0%)	245 (52.9%)	398 (50.9%)	.17
RR > 18	190 (59.6%)	287 (62.0%)	477 (61.0%)	.50
Encounter location				
ED	249 (78.1%)	305 (65.9%)	554 (70.8%)	<.001
In‐hospital service	38 (11.9%)	103 (22.2%)	141 (18.0%)	
Transfer	27 (8.5%)	36 (7.8%)	63 (8.1%)	
Clinic	5 (1.6%)	19 (4.1%)	24 (3.1%)	

*Note*: Data presented as median [Q1–Q3] or count (%).

Abbreviations: BMI, body mass index; CAD, coronary artery disease; CHF, congestive heart failure; COPD, chronic obstructive pulmonary disease; ED, Emergency Department; HR, heart rate; PE, pulmonary embolism; RR, respiratory rate; SBP, systolic blood pressure; SpO_2_, oxygen saturation.

### 3.2 Diagnostic testing and risk categories

Imaging and laboratory findings are shown in Table 2. Cardiac biomarkers (troponin and BNP) and radiographic images were ordered with similar frequency with no difference in the frequency of abnormal results between groups. Black patients were more likely to have right ventricle (RV) hypokinesis (45.3 vs. 34.8%, *p* = .016) and elevated right ventricular systolic pressure (RVSP) (Table 2). There was no significant difference in risk stratification, as defined by the 2019 European Society of Cardiology (ESC) guidelines for acute PE, between Black and White patients (Table S1).

**Table 2:** Diagnostic and radiographic tests ordered by race.

	Black (*N* = 319)	White (*N* = 463)	Total (*N* = 782)	*p* value
Cardiac biomarkers				
Troponin ordered	226 (70.8%)	304 (65.7%)	530 (67.8%)	.13
Troponin positive ( > 0.1 ng/mL)	42/226 (18.6%)	44/304 (14.5%)	86/530 (16.2%)	.20
pro‐BNP ordered	174 (54.5%)	242 (52.3%)	416 (53.2%)	.53
pro‐BNP positive ( > 850 pg/mL)	93/174 (53.4%)	144/242 (59.5%)	236/416 (57.0%)	.22
Diagnostic radiographic tests ordered				
LE ultrasound	214 (67.1%)	316 (68.3%)	530 (67.8%)	.73
Echocardiogram	212 (66.5%)	313 (67.6%)	525 (67.1%)	.76
Computed tomography	287 (90.0%)	429 (92.7%)	716 (91.6%)	.19
Angiography	2 (0.6%)	0 (0.0%)	2 (0.3%)	.17
V/Q scan	30 (9.4%)	31 (6.7%)	61 (7.8%)	.17
Computed tomography test results				
	*N* = 287	*N* = 429	*N* = 716	
Saddle	10 (3.5%)	27 (6.3%)	37 (5.2%)	.096
Main PA	86 (30.0%)	104 (24.2%)	190 (26.5%)	.089
Lobar	145 (50.5%)	203 (47.3%)	348 (48.6%)	.40
Sub/segmental	191 (66.6%)	306 (71.3%)	497 (69.4%)	.17
RV/LV ratio >0.9	44 (77.2%)	46 (64.8%)	90 (70.3%)	.13
Echocardiogram test results				
	*N* = 212	*N* = 313	*N* = 525	
RV enlargement (basal diameter >4.1 cm)	102 (48.1%)	131 (41.9%)	233 (44.4%)	.16
RV hypokinesis	96 (45.3%)	109 (34.8%)	205 (39.0%)	.016
RVSP, mmHg				.039
<35	122 (57.5%)	194 (62.0%)	316 (60.2%)	
35–45	28 (13.2%)	54 (17.3%)	82 (15.6%)	
46–60	29 (13.6%)	40 (12.8%)	69 (13.1%)	
>60	33 (15.6%)	25 (8.0%)	58 (11.0%)	

Abbreviations: BNP, B‐type natriuretic peptide; LE, lower extremity; LV, left ventricle; PA, pulmonary artery; RV, right ventricle; RVSP, right ventricular systolic pressure; V/Q, ventilation perfusion.

### 3.3 Advanced therapies

The use of advanced therapies is depicted in Table 3. Black patients with acute PE received systemic tissue plasminogen activator (tPA) more frequently than White patients with acute PE (3.1% vs. 1.1%, *p* = .040) (Table 3). There was no difference in the use of catheter‐directed thrombolysis (CDT) or extracorporeal membrane oxygenation (ECMO) between groups (Table 3).

**Table 3:** Pulmonary embolism therapeutics during index hospitalization by race.

	Black (*N* = 319)	White (*N* = 463)	Total (*N* = 782)	*p* value
Advanced therapies				
Systemic tPA	10 (3.1%)	5 (1.1%)	15 (1.9%)	.040
CDT	12 (3.8%)	18 (3.9%)	30 (3.8%)	.93
Embolectomy	1 (0.3%)	5 (1.1%)	6 (0.8%)	.41
ECMO	2 (0.6%)	3 (0.6%)	5 (0.6%)	>.99
Anticoagulation				
LMWH	62 (19.4%)	141 (30.5%)	203 (26.0%)	.001
Warfarin	75 (23.5%)	56 (12.1%)	131 (16.8%)	<.001
DOAC	152 (47.6%)	222 (47.9%)	374 (47.8%)	.93

Abbreviations: CDT, catheter‐directed therapy; DOAC, direct oral anticoagulant; ECMO, extracorporeal membrane oxygenation; LMWH, low molecular weight heparin; tPA, tissue plasminogen activator.

### 3.4 Hospital utilization and mortality

Hospital utilization, intensive care unit (ICU) utilization and discharge destination are shown in Table 4. Black patients had significantly longer hospital LOS compared with White patients (median [Q1–Q3]: 5 [3–9] vs. 4 [2–9] days, *p* = .007). There was no difference in discharge destination (*p* = .46) among patients who survived or all‐cause mortality (3.1 vs. 3.9%, *p* = .58) between Black and White patients, respectively (Table 4). While anticoagulant use was similar (90.9 vs. 90.5%, *p* = .85), the type of anticoagulant did differ between Black and White patients. Warfarin was prescribed at discharge more often in Black compared to White patients (23.5 vs. 12.1%, *p* < .001), while White patients were more often discharged on low‐molecular‐weight heparin (30.5 vs. 19.4%, *p* = .001). No difference was found in the use of direct oral anticoagulants between Black and White patients (47.6 vs. 47.9%).

**Table 4:** Mortality from acute pulmonary embolism and hospital utilization by race.

	Black (*N* = 319)	White (*N* = 463)	Total (*N* = 782)	*p* value
Discharge status				
Alive	309 (96.9%)	445 (96.1%)	754 (96.4%)	.58
Deceased	10 (3.1%)	18 (3.9%)	28 (3.6%)	
Deceased or hospice	27 (8.5%)	43 (9.3%)	70 (9.0%)	.69
Intensive care unit utilization				
Number admitted	89 (27.9%)	116 (25.1%)	205 (26.2%)	.37
*ICU LOS (days)	2.8 (1.2–6.5)	3.1 (1.5–7.0)	2.9 (1.3–6.8)	.41
Intubation	28 (8.8%)	41 (8.9%)	69 (8.8%)	.97
Hospital utilization				
*Hospital LOS (days)	5.0 (3‐9)	4.0 (2‐9)	5.0 (3‐9)	.007
Discharge locations				
	*N* = 309	*N* = 445	*N* = 754	.46
Home	233 (75.4%)	350 (78.6%)	583 (77.3%)	
Hospice	17 (5.5%)	25 (5.6%)	42 (5.6%)	
Rehabilitation	48 (15.5%)	63 (14.2%)	111 (14.7%	
LTAC	4 (1.3%)	2 (0.4%)	6 (0.8%)	
Transfer	7 (2.3%)	5 (1.1%)	12 (1.6%)	

Abbreviations: ICU, intensive care unit; LOS, length of stay; LTAC, long‐term care facility.

* Intensive care unit (ICU) and hospital length of stay (LOS) computed as time to discharge or death, whichever occurred first. Data presented as median (Q1–Q3) or count (%).

Multivariable models adjusted for baseline risk factors imbalanced between race groups (Table 5) found that warfarin use (OR: 1.88, 95% CI: 1.24–2.86, *p* = .003) and hospital LOS (IRR: 1.18, 95% CI: 1.04–1.35, *p* = .011) remained significantly higher for Black compared with White patients. No differences were found with regard to ICU admittance, ICU LOS, or the combined outcome of in‐hospital death or discharge to hospice.

**Table 5:** Univariable and multivariable regression models for race.

	Reference	Univariable	Multivariable*
Dichotomous outcome		OR (95% CI)	OR (95% CI)
Deceased or hospice	Black vs. White	1.11 (0.67–1.83)	1.22 (0.70–2.12)
ICU admission	Black vs. White	1.16 (0.84–1.60)	1.12 (0.78–1.59)
Warfarin use	Black vs. White	2.23 (1.53–3.27)	1.88 (1.24–2.86)
Continuous outcome		IRR (95% CI)	IRR (95% CI)
ICU LOS	Black vs. White	0.97 (0.74–1.29)	1.01 (0.75–1.35)
Hospital LOS	Black vs. White	1.14 (1.00–1.29)	1.18 (1.04–1.35)

Abbreviations: CI, confidence interval; ICU, intensive care unit; IRR, incidence rate ratio; LOS, length of stay; OR, odds ratio.

* Adjusted for age, body mass index, hypertension, congestive heart failure, diabetes, kidney disease, cancer, and prior surgery/trauma.

## 4 DISCUSSION

In this study, we compared in‐hospital outcomes among Black and White patients admitted with acute PE. We found no difference in the composite endpoint of all‐cause mortality during index hospitalization for PE and discharge to hospice care. Black patients had significantly longer hospitalizations even after adjusting for confounders, but similar utilization of critical care resources. Malignancy or recent surgery was more common amongst White patients with acute PE, while Black patients more often had cardiometabolic risk factors. Imaging studies and cardiac biomarkers were utilized similarly in both groups.

Malignancy, surgery, trauma, and inpatient hospitalization are traditional risk factors for acute PE, but have historically been assessed in predominantly White populations.^18^, ^19^, ^20^, ^21^, ^22^ In our cohort, these well‐established risk factors were more likely to be present among White patients, whereas Black patients more frequently had heart failure, diabetes mellitus, and kidney disease. It is well established that Black patients experience disparate care for many common health conditions, including routine cancer screenings and elective surgical procedures, and so it is possible the absence of known VTE risk factors may be the byproduct of this inequity.^23^, ^24^, ^25^, ^26^ We did not capture malignancy diagnosis during or after index hospitalization, and it is possible the malignancy rate is artificially low in the Black population due to delayed diagnosis, with PE as the sentinel event prompting diagnostic work up. However, our findings build on a trend seen in several other studies that acute PE risk factors may be different in Black patients.^27^, ^28^ While there is some evidence to suggest Black patients with acute PE may have underappreciated thrombotic risk due to higher levels of hemostatic factors and endothelial markers, including factor VIII, von Willebrand factor, plasmin antiplasmin complex, and d‐dimer, despite lower incidence of inherited thrombophilia, an exact biologic explanation is unestablished.^27^ Regardless of the explanation, frontline clinicians must be aware of the different risk profiles and understand that historic risk stratification scores may not perform as well in minority populations.

In our cohort, there was no difference in in‐hospital mortality between Black and White patients with acute PE. This is a divergence from prior studies and likely reflects several unique aspects of PE care that facilitate healthcare equity.^12^, ^29^, ^30^ First, CT utilization has dramatically risen in the last decade and offers the advantage not only of being widely available in emergency departments but also allows for RV assessment at the time of PE diagnosis.^31^, ^32^ Additionally, troponin and BNP assessment of the undifferentiated patient presenting to the emergency department have become standard in the initial work‐up of chest pain and shortness of breath.^33^ Many emergency departments, including our own, have adopted algorithms to guide the workup of undifferentiated patients and those suspected of having PE.^34^, ^35^ The widespread implementation of these tools can counteract implicit bias and makes PE care distinct from other fields that still rely on clinician assessment to determine candidacy for therapies.^4^

While advanced therapies for the management of PE have emerged in recent years,^36^, ^37^, ^38^, ^39^, ^40^ their utilization was low in the present cohort, limiting the ability to detect a disparity. Less than 5% of all patients received catheter‐based therapies or ECMO. Whereas cardiovascular diseases such as heart failure have successfully implemented widespread guideline‐directed medical therapies and advanced mechanical circulatory support options, data supporting optimal patient selection for catheter‐based therapies is limited and anticoagulation remains the mainstay of PE management. We did observe a disproportionate use of warfarin therapy amongst Black patients compared to other anticoagulants. It is possible that the initiation of warfarin and concurrent need for bridging therapy contributed to the longer hospital LOS observed in the Black population. DOAC therapy, however, does not require this bridging period, and has been associated with a reduced hospital length of stay.^41^ Additionally, over 30% of White patients were treated with low‐molecular‐weight heparin, an observation that may reflect the higher rate of malignancy in this group. While data on insurance status or medication co‐payment was not available for this cohort, these potential confounders may not fully explain these trends.

This study had several limitations that are important to consider. First, this single‐center study only captures outcomes from the index hospitalization over an 18‐month period. Both active and remote malignancy were included in cancer history, so the impact of malignancy under active treatment is incompletely quantified. The relationship between race and cardiovascular outcomes is complex and often confounded by socioeconomic status, access to health care, and other factors, for which we were unable to account. However, it is less likely that this would have been a factor in in‐hospital outcomes, which are less frequently influenced by insurance status and patient cost.

## 5 CONCLUSION

In this large cohort of patients hospitalized for acute PE, there were no differences in mortality, discharge to hospice, or ICU utilization between Black and White patients, but Black patients were younger with more cardiometabolic risk factors than White patients, had longer hospitalizations, and more warfarin prescription. While no significant mortality or treatment disparity was found, large‐scale studies powered to assess for differences in advanced therapy allocation are still needed to help clinicians better identify patients who stand to benefit from these interventions. Further investigation into racial differences in thromboembolic risk profiles is needed.

## CONFLICTS OF INTEREST STATEMENT

Manesh R. Patel: Advisory Board/Consultant: Bayer, Janssen, Novartis, Medscape, Research Grants: Bayer, Janssen, Novartis, NHBLI. All the remaining authors declare no conflicts of interest.

## Supporting information

Supporting information.

## Data Availability

Research data are not shared.
