# Supplementary material for: Association of race and in‐hospital outcomes following acute pulmonary embolism: A retrospective cohort study
Source: Clin Cardiol. 2023 May 31;46(7):768–76. doi: 10.1002/clc.24055 (PMC10352978; doi:10.1002/clc.24055)
Supplement: Supplementary file 1 — Supporting information. [file CLC-46-768-s001.docx]

Supplementary Table 1. European Society of Cardiology risk category distribution by race

|  | **Black (N=319)** | **White (N=463)** | **Total (N=782)** | **P value** |
| --- | --- | --- | --- | --- |
| Low | 169 (53.0%) | 254 (54.9%) | 423 (54.1%) | 0.52 |
| Intermediate Low | 59 (18.5%) | 97 (21.0%) | 156 (19.9%) |  |
| Intermediate High | 70 (21.9%) | 83 (17.9%) | 153 (19.6%) |  |
| High | 21 (6.6%) | 29 (6.3%) | 50 (6.4%) |  |
